# The Effects of Different Anticavity Agents and Er:YAG Laser Usage on Enamel Surface Microhardness

**DOI:** 10.3290/j.ohpd.a43893

**Published:** 2020-07-04

**Authors:** Yiğit Kaan Polat, Nurcan Ozakar Ilday

**Affiliations:** a Research Assistant, Department of Restorative Dentistry, Faculty of Dentistry, Atatürk University, Erzurum, Turkey. Research, conception and study design; data acquisition and interpretation; preparation and final approval of the manuscript.; b Associate Professor, Department of Restorative Dentistry, Faculty of Dentistry Atatürk University, Erzurum, Turkey. Research, conception and study design; data acquisition and interpretation; preparation and final approval of the manuscript.

**Keywords:** bioactive glass, casein phosphopeptide amorphous calcium phosphate, demineralisation, Er:YAG laser, remineralisation, titanium tetrafluoride

## Abstract

**Purpose:**

The purpose of this study was to perform an in vitro evaluation of the protective effects of anticavity agents applied to enamel, by themselves and in combination with Er:YAG.

**Materials and Methods:**

In this study 150 extracted third molars were used. Measurements were made using DIAGNOdent, and samples with scores of 0–13 were included in the study. These were divided into 15 groups (n = 20). Use of the agents sodium fluoride (NaF), tricalcium phosphate (Ca_3_PO_4_), titanium tetrafluoride (TiF_4_), Tooth Mousse (CPP-ACP), MI Paste Plus (CPP-ACP), and NovaMin (bioactive glass), individually and then in combination with Er:YAG laser, was assessed based on their effects on microhardness values. After treatment, the groups were exposed to a demineralisation solution. Statistical analyses were conducted using SPSS 20.0 package software.

**Results:**

The highest test result value was observed in the TFL (TiF_4_/Er:YAG laser) group. Statistically significant differences were determined among all the groups (p <0.05). When the groups in which the agents were applied alone were compared with those involving combined use of Er:YAG laser, combined use provided significantly higher microhardness values, with the exception of the TML group (Tooth Mousse/Er:YAG laser) (p <0.05).

**Conclusion:**

Within the limits of this study, the combined use of remineralisation agents and Er:YAG laser elicited better results than using the agents alone. The most effective remineralisation agent was TiF_4_/Er:YAG laser, which may be considered an alternative method for protecting the enamel against demineralisation.

As our understanding of the pathogenesis of cavities and methods of protection against cavities has improved in recent years, procedures aimed at preventing demineralisation in the enamel surface and activating remineralisation have acquired increasing importance. Invasive operations in therapeutic approaches have been replaced by minimally invasive and even non-invasive strategies.^6,20^

However, although products that contain fluoride and topical fluoride applications achieve statistically significant reductions in cavities, there is still a very large population affected by cavities.^12^ Binding of fluoride to metal ions is recommended in order to increase intake from fluoride agents by dental tissues. The glaze-like layer created on the surface by the titanium and fluoride combination TiF_4_ is reported to strengthen the tooth surface.^1,28,29^

In addition to known protective application protocols, systems that contain calcium phosphate have been developed and presented for clinical application in recent years. Casein phosphopeptide amorphous calcium phosphate (CPP-ACP), obtained from milk casein, is one such product. In vitro and in situ studies have reported positive results in the professional use of CPP-ACP.^5,11,26^

Previous studies have investigated the characteristics of the agent tricalcium phosphate in the prevention of cavities and improvement of remineralisation. Studies of products containing bioactive glass have shown that these agents are potentially beneficial in terms of remineralisation and increasing anticavity resistance.^1,7,15^

DIAGNOdent is one of the tools that are frequently used for cavity detection. Its working mechanism is based on the fact that healthy surrounding tissues and cavity tissue absorb light differently. Inclusion of healthy samples in studies using this device for scoring is important in terms of standardisation of results.^23-25^

Due to technological advances, hard tissue laser applications have begun to be adopted as a clinical approach. Studies have reported that Er:YAG laser, one type of hard tissue laser, strengthens the enamel against acid attacks and prevents mineral loss.^3,18^ However, studies of the effectiveness of current anticavity agents in combination with laser treatments are limited.

The objectives of this study were:

1. To analyse the activity of various anticavity agents applied to teeth using a microhardness analysis method.
2. To determine the activities of Er:YAG laser, a hard tissue laser, used either alone or in combination with these agents in cavity formation.

## Materials and Methods

This study was approved by the Atatürk University Faculty of Dentistry ethical committee (No. 14/05, dated 27.10.2015).

One hundred and fifty human wisdom teeth were used in order to obtain standard enamel surfaces. Tissue residues were removed using periodontal curette, a polishing prophy cup and pumice. A stereomicroscope (×30 magnification) was used to check whether there was any developmental defect on the buccal and lingual surfaces. After cleaning, the teeth were left in a 0.1% thymol solution at +4ºC for disinfection. After 1 week, the teeth were removed from the thymol solution and kept in bidistilled water through the study. Measurements were taken using a laser fluorescence device (DIAGNOdent Pen, KaVo, Zürich), and those with healthy scores (0–13) were included in the study.

After the roots had been removed with a diamond separator under water cooling, the crown parts were divided into two sections in a mesiodistal direction. A smooth and regular surface was obtained by sanding with 600–grid silicon carbide. Rectangular areas 4 × 5 mm in size were left open on the buccal and lingual surfaces, while all remaining areas were covered with two layers of durable nail polish. The initial microhardness values of samples with no cracks or defects on their surface were measured by applying a 100 N force for 15 min using a microhardness testing device (Fm 800e Future Tech, Tokyo, Japan).

An acetate frame capable of passing through the four corners of the 3 × 4 mm^2^ rectangular windows created on the enamel surface was prepared for measurement, and two points were drilled. This enabled measurements to be repeated from the same points after demineralisation. Measurements were taken at different time intervals using the DIAGNOdent Pen (KaVo Dental, LakeZurich, IL, USA) device. The first measurement was taken on the healthy enamel before starting the demineralisation process, and the other measurements were taken after 9 days, when the demineralisation process had been completed. The scores were obtained in the measurements collected by the DIAGNOdent Pen (KaVo Dental) device.^24,25^ As defined by the manufacturer, scores of 0–13 represent healthy enamel, scores of 14–20 represent initial demineralisation, scores of 21–29 represent advanced demineralisation, and scores of 30 or more represent cavities. These measurements permitted determination of the initial enamel lesions. Samples with scores of 18–20 after demineralisation were included in the study.

Samples were divided into 15 groups, depending on the anticavity agent and laser application employed (positive control = demineralisation only, negative control = no application) (n = 20).

After the anticavity agents in the non-laser groups (Group N [NaF, 4%], Group TM [Tooth Mousse], Group TF (TiF_4_, 4%), Group M [MI Paste Plus], Group T [tricalcium phosphate, 4%], and Group BG [bioactive glass NovaMin]) had been applied such as to cover the enamel surface, these were left on the surface for 4 min (Table 1). Excess material was removed from the sample surfaces using a brush. In the laser treatment groups, Er:YAG laser (Fotona Fidelis III AT, Slovenia, 2.94 µm wavelength) was applied to the 4 × 5 mm rectangular surfaces of the samples’ buccal or lingual central thirds for 30 sec in non-contact mode, 0.5 mm distance, using an air-water spray with the help of the device’s sapphire-tip hand-held piece (1000 μs, 80mJ, 10 Hz, 1.3 tip diameter).^21^ The application was performed using a sweeping motion. After laser treatment, the surfaces were exposed to the various anticavity agents for 4 min. With the exception of Group K (negative control), the other 14 groups were placed in a pH cycle that corresponded to clinical conditions, and we attempted to create cavities. This model varied in the form of 5-, 9- and 14-day periods. The pH cycle model was created as described previously elsewhere.^9^ Samples were stored in individual glass bottles in a demineralisation solution containing 2.0 mmol/L Ca, 2.0 mmol/L P, and 0.075 mol/L acetate (pH 4.3) for 8 h at 37°C. The samples were then removed from this solution and washed with bidistilled water. For the next 16 h, the samples were kept in 1.5 mmol/L Ca, 0.9 mmol/LP, and 150 mmol/L KCl, and 20 mmol/L cacodylate buffer at 37ºC, and in a remineralisation solution (pH 7.0). The pH cycle was maintained for 9 days. Solutions were changed every 3 days to prevent saturation.

**Table 1:** Anticavity agents and their contents

Anticavity agents	Manufacturer	Lot	Content
Tooth Mousse	GC, Japan	151017S	CPP-ACP
MI Paste Plus	GC, Japan	150413S	CPP-ACP, Fluoride
Novamin	Sensodyne, UK	245D	Bioactive glass, fluoride
Titanium tetra fluoride (4%)	Sigma Aldrich, USA	MKBS9207V	Titanium, fluoride
Calcium phosphate (4%)	Sigma Aldrich, USA	BCBR7535V	Tricalcium phosphate, fluoride
Sodium fluoride (4%)	Sigma Aldrich, USA	MKBW6844V	Sodium, fluoride

**Fig 1:**
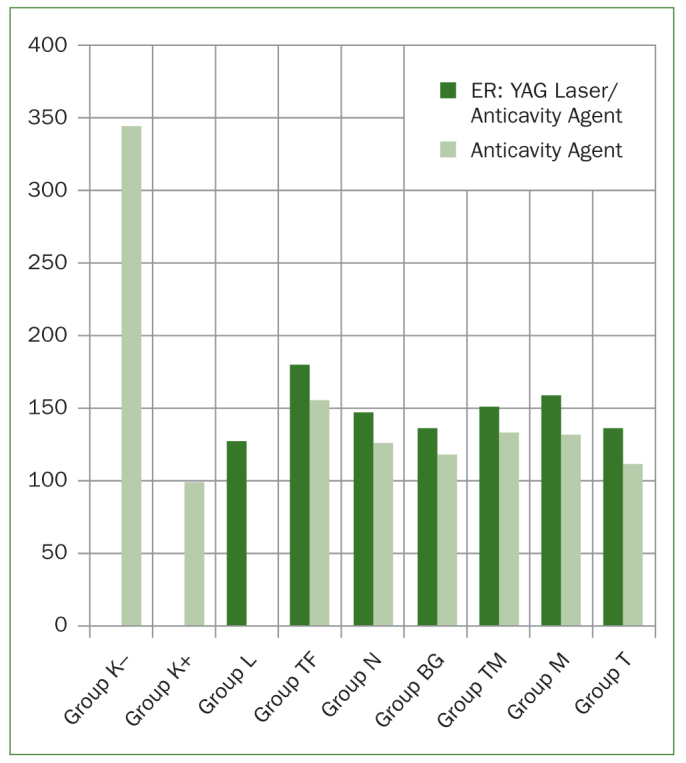
Comparison of anticavity agent and Er:yag laser/anticavity agent usage for each group.

**Fig 2:**
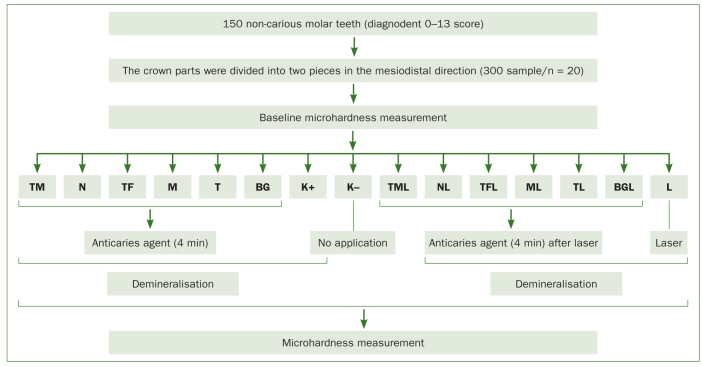
Schematic representation of experimental phases.

The microhardness of all samples was measured by applying a 100-µ Newton force for 15 s using a microhardness testing device with a Vickers tip. The microhardness values obtained from the samples at the beginning of and after treatment were analysed on IBM SPSS 20.0 software. One-way analysis of variance (ANOVA) and Duncan’s multiple comparison tests were used to compare the microhardness values of the groups at the beginning of and after treatment. The paired t test was used to compare intragroup hardness values before and after treatment in each group. In order to determine the activity of the laser treatment, the independent samples t test was applied to compare data for the agents used by themselves and those for the agents used in combination with laser. The results were interpreted at a statistical significance level of p <0.05 and at a 95% confidence interval (Figs 1 and 2).

## Results

Table 2 shows the groups’ microhardness values (mean ± standard deviation) obtained before and after treatment.

**Table 2:** Means and standard deviations values of microhardness (VHN) data obtained from groups baseline and after application

Groups	Baseline	After application	p*
Group K– (negative control)	350.63 ± 53.00^a^	343.85 ± 52.84^a^	0.475
Group K+ (positive control)	343.25 ± 36.20^a^	98.99 ± 4.95^ı^	0.000
Group L (Er:YAG Laser)	341.10 ± 37.14^a^	126.58 ± 15.64^fgh^	0.000
Group TF (TiF_4_)	332.31 ± 42.80^a^	155.17 ± 15.01^c^	0.000
Group TFL (TiF_4_/Er:YAG Laser)	333.95 ± 40.34^a^	179.15 ± 12.56^b^	0.000
Group N (NaF)	335.01 ± 48.36^a^	126.08 ± 6.7^fgh^	0.000
Group NL (NaF/Er:YAG Laser)	349.81 ± 45.65^a^	147.00 ± 13.35^cde^	0.000
Group BG (Bioactive glass)	344.81 ± 29.12^a^	117.68 ± 6.73^gh^	0.000
Group BGL (Bioactive glass/Er:YAG Laser)	318.42 ± 34.30^a^	136.08 ± 7.12^def^	0.000
Group TM (Tooth Mousse)	352.04 ± 41.61^a^	132.97 ± 16.45^efg^	0.000
Group TML (Tooth Mousse/Er:YAG Laser)	347.44 ± 44.19^a^	151.66 ± 18.59^cd^	0.000
Group M (MI Paste Plus)	330.5 ± 40.82^a^	131.00 ± 16.29^efg^	0.000
Group ML (MI Paste Plus/Er:YAG Laser)	340.74 ± 46.54^a^	158.64 ± 20.44^c^	0.000
Group T (Tricalcium phosphate)	341.81 ± 45.92^a^	112.6 ± 11.28^hı^	0.000
Group TL (Tricalcium phosphate/Er:YAG Laser)	337.16 ± 37.72^a^	135.84 ± 9.53^def^	0.000

The different lowercase superscript letters in the columns are statistically different. p <0.05; one-way ANOVA, Duncan; p*, paired t test.

The highest microhardness value was determined in Group TM (Tooth Mousse, 352.04 ± 41.61.) The lowest value was observed in Group BGL (NovaMin/Er:YAG Laser, 318.42 ± 34.30). However, the differences between the groups were not statistically significant (p >0.05) (Table 2).

After the application of anticavity agent/laser, the highest microhardness value was observed in Group TFL (TiF_4_ /Er:YAG Laser). Statistically significant differences were determined among the all groups (p <0.001) (Table 2).

Microhardness values at baseline and after application of anticavity agents differed significantly (p <0.001), except for in Group K (negative control) (p <0.001) (Table 2).

 A statistically significant difference was observed between the non-laser and the laser/anticavity agent groups (p <0.001) (Table 3).

**Table 3:** Comparison of microhardness values (VHN) of laser-treated and non-laser-treated groups (independent samples t test)

Groups	Baseline	p	After application	p
Group TF	332.31 ± 42.80	0.927	155.17 ± 15.01	0.001
Group TFL	315.77 ± 16.18		179.15 ± 12.56	
Group N	335.01 ± 48.36	0.469	126.08 ± 6.70	0.000
Group NL	349.81 ± 45.65		147.00 ± 13.35	
Group BG	344.81 ± 29.12	0.066	117.68 ± 6.73	0.000
Group BGL	318.42 ± 34.30		136.08 ± 7.12	
Group TM	352.04 ± 41.61	0.804	143.02 ± 10.63	0.021
Group TML	347.44 ± 44.19		151.66 ± 18.59	
Group M	330.75 ± 40.82	0.598	131.70 ± 16.29	0.003
Group ML	340.74 ± 46.54		158.64 ± 20.44	
Group T	340.74 ± 46.54	0.798	112.26 ± 11.28	0.000
Group TL	337.16 ± 37.72		135.84 ± 9.53	

## Discussion

Several studies aiming to prevent cavity formation have been conducted by developing protective agents with different mineral contents and application methods.^4,8,10,14,17^ Our study was intended to assess different agents with anticavity properties and Er:YAG laser, with a known protective effect against cavities and used as a hard tissue laser, based on enamel microhardness values.

Erbium laser has a wavelength of 2.94 μm, which corresponds exactly to the absorption point of water and the level absorbed by hydroxyapatite, and is therefore the most frequently employed laser for hard tooth tissues.^7,10^ In order to use the anticavity properties of Erbium lasers, they may be employed under the ablation threshold value.^2,6,16^

The first topically applied fluoride compound was sodium fluoride (NaF). Preparates with NaF content have neutral pH. Several studies have reported that sodium fluoride exhibits better cavity prevention than monofluorophosphate.^7,12,13,30^ In agreement with previous studies, we observed a statistically significant difference between the NaF group (Group N) and Group K+. We attributed this to the CaF_2_ crystals that formed on the surface strengthening the surface against acid attacks and preventing demineralisation.

Mundorff et al^19^ reported the formation of an organo-metallic complex between the organic part of the enamel and titanium as a result of local TiF_4_ application, and that this caused the formation of a glaze-like, acid-resistant layer on the surface. Our microhardness measurements showed that the group in which TiF_4_ was applied by itself, Group TF, was significantly more effective in preventing demineralisation than Group K+ (positive control) (p <0.001). We attributed this to Ti-forming organo-metallic bonds with other surface ions. The agent TiF_4_ thus exhibited more effective resistance than other remineralisation agents (p <0.001).

Tricalcium phosphate and fluoride bind to each other on the tooth surface at neutral pH to form calcium fluoride. This accelerates the remineralisation process.^1^ A previous study reported that agents with TCP content are effective in protecting teeth and achieve high levels of remineralisation.^15^ In the present study, the group in which an agent with tricalcium phosphate content was applied, Group T, differed significantly from Group K+ in terms of microhardness values (p <0.001). However, compared to other groups in terms of resistance to demineralisation, Group T exhibited low values, similar to those of Group BG (p >0.05).

The effects of CPP-ACP on the teeth are achieved with calcium and phosphate reservoirs. Several studies have reported the remineralisation effects of CPP-ACP on tooth enamel.^4,14,22^ Yaasaei et al^29^ showed that the use of CPP-ACP in combination with the Er:YAG laser was effective in reducing demineralisation. In this study, Group TM and Group M exhibited similar postdemineralisation microhardness values, which were significantly higher than those in Group K+ (positive control) (p <0.001). Lata et al^14^ investigated the use of fluoride, CPP-ACP and a combination of the two in terms of remineralisation effects as reflected by microhardness values. Enamel surfaces treated with fluoride had a higher microhardness value than those treated with CPP-ACP. However, the difference was not statistically significant. Similarly, we observed no statistically significant difference between Group N, Group TM and Group M in terms of microhardness values (p >0.05). This may have been due to the method and duration of administration of CPP-ACP. Further studies are now needed to clarify this issue.

In their study of the effects of bioactive glass and sodium fluoride on initial-stage cavity lesions, Dlamanti et al^7^ reported that bioactive glasses may represent an alternative to fluoride as remineralisation agents. Postdemineralisation microhardness values were significantly higher in Group BG than in Group K+. However, when Group BG was compared to those groups in which other agents were applied, it exhibited low values, similarly to Group T, in which tricalcium phosphate was used. Bevilacqua et al^4^ stated that different fluencies of Er:YAG (1.8 J/cm^2^ and 0.9 J/cm^2^) reduced acid dissolution and increased fluoride uptake, and suggested that it may be used as an alternative method in preventive dentistry.

Combined use of laser and fluoride provides a synergic effect in reducing enamel dissolvability. Topical fluoride application before or after laser application reduces the rate of dissolution of the enamel structure in acidic solutions and increases the fluoride intake rate.^27^

If the surface is treated with laser before or after application, the surface melts and recrystallises in form of fluorapatite minerals.^30^ The groups in which anticavity agents were used in combination with laser (groups TML, ML, NL, BGL and TL) had significantly higher microhardness values than those of the groups in which no laser treatment was involved (p <0.05).

Magalhaes et al^17^ investigated the effects of NaF and TiF_4_ agents used in combination with Nd:YAG on laser enamel erosion and reported that the group in which TiF_4_ was applied created higher resistance to erosion in comparison to the other groups, and that the use of TiF_4_ by itself or in combination with laser resulted in no statistically significant differences. Group TFL exhibited significantly different postdemineralisation microhardness values to those of Group TF in our study.

When Group M and Group TM were compared to Group ML and Group TML in terms of microhardness values, the groups in which combined laser treatment was not applied exhibited lower resistance to demineralisation. Our review of the literature revealed very little research on this topic, and further studies are needed.

We encountered no information in the literature regarding the use of tricalcium phosphate in combination with laser. Based on the microhardness values obtained in our study, Group TL provided better results in terms of resistance to demineralisation in comparison to Group T. The postdemineralisation microhardness values in Group BGL were higher than those in Group BG. In terms of resistance to cavity formation, we encountered no previous studies investigating the use of bioactive glass or tricalcium phosphate in combination with lasers.

## Conclusion

Within the limitations of this study we concluded that:

1. In comparison to the positive control group, all treatment groups were effective in preventing demineralisation to a statistically significant extent (p <0.05).
2. When laser treatment alone was compared to the groups in which anticavity agents were applied without laser treatment, the groups with anticavity agents achieved higher microhardness values (p <0.05).
3. Combined application with Er:YAG laser treatment provided more effective protection than applying anticavity agents by themselves (p <0.05).
4. All agents used to prevent enamel surface demineralisation (CPP-ACP, NaF, TiF_4_, TCP and NovaMin) were found to be effective. The most effective agent was TiF_4_.
